# Coach’s Perception of Parent’s Attitudes in School-Age Sports

**DOI:** 10.3390/ijerph182010701

**Published:** 2021-10-12

**Authors:** Santos Villafaina, Eugenio Merellano-Navarro, Juan M. García-Ceberino

**Affiliations:** 1 Physical Activity and Quality of Life Research Group (AFYCAV), University of Extremadura, 10003 Caceres, Spain; svillafaina@unex.es; 2 Grupo de Investigación en Educación Física, Salud y Calidad de Vida (EFISAL), Universidad Autónoma de Chile, Talca 3460000, Chile; 3 Faculty of Humanities and Social Sciences, University of Isabel I, 09003 Burgos, Spain; juanmanuel.garcia.ceberino@ui1.es; 4 Optimization of Training and Sports Performance Research Group (GOERD), University of Extremadura, 10003 Caceres, Spain

**Keywords:** communication, competition attitude, environment, institution type, sex

## Abstract

The coach-parent relationship can significantly influence the sport participation, personal development, and sports performance of young athletes. This study aimed to investigate the coach’s perception of parent’s attitudes regarding competition attitude, the communication with the coach, and the environment during training sessions, taking into account the coaches’ sex, type of institution, or sport modality grouped into individual and collective sports. For this, a total of 159 coaches (22 females and 137 males) responded to the Questionnaire for Sports Coaches about their Opinion on the Attitudes of Fathers and Mothers in Sports (CTPMD). It analyses three factors: (1) competition attitude, (2) communication, and (3) environment. A Confirmatory Factor Analysis (CFA) was necessary to assess the model’s goodness of fit on each factor and to calculate the Cronbach’s Alpha, the average variance extracted, and the composite reliability to analyze the instrument reliability. Descriptive and inferential analysis was performed through the Mann-Whitney U and Rosenthal r statistical tests. The results showed significant differences in the competition attitude factor regarding type of institution (*U* = 1964.500; *p*-value < 0.001; *r* = 0.31), in favour of public institutions. There were also significant differences in the communication factor according to the coaches’ sex (*U* = 1112.000; *p*-value = 0.04; *r* = 0.16), with females’ coaches perceiving worse communication with parents than male coaches. This could be relevant because it negatively influences adherence to physical activity in children and adolescents, and therefore, their health.

## 1. Introduction

According to the World Health Organization [1], physical activity enrolment during childhood is of great importance to children’s health and correct development. In this regard, physical activity benefits can improve social [2], mental [3], and physical health [4], decreasing the risk of chronic diseases [5] such as obesity [6,7]. Nowadays, these health benefits are especially relevant since the COVID-19 pandemic has increased the level of physical inactivity and sedentary behaviours in children and adolescents [8], leading to a decrease in the health-related quality of life [9].

Sport is one of the main options for children who invest their leisure time in an active purpose [10,11]. The National Alliance for Youth Sports estimates that approximately 65% of children worldwide are enrolled in sports activities. Sport is usually organized, competitive, and could be played in a team or individual [12]. In Europe, different institutions offer different options, such as clubs or public institutions that offer sport modalities as a leisure option for children. In this regard, differences between clubs and public institutions could be the performance-oriented of clubs with a more educational goal of public institutions. Adherence to any of these options is quite important for children and adolescents since it has been demonstrated that those children who actively participate in these programs obtain positive effects on children’s health, education, and behaviour [13]. Furthermore, apart from the physical benefits of exercise, the social support and acceptance that being part of a team can provide relevant psychosocial benefits such as an increased resilience in terms of the negative processes that push teenagers toward suicide [14,15].

During children’s sports practice, three important social agents are involved: parents, coaches, and peers, since they can significantly influence their sports participation, personal development, and sports performance [16]. In this regard, these three figures (parents, coaches and peers) contribute to the motivational climate that young athletes experience [17]. In this regard, parents allow them to experience various sports, taking on a monitoring and support role, while coaches should provide them with positive experiences during sports practice [18]. For these reasons, coaches assume an important educational and socialization function, and parents play an essential motivating role in their children [19]. The motivational climate of parents can influence how children understand, perceive and react to achievement such as competitions or training [20].

Nevertheless, parental attitude in the sport context could condition the children’s sports practice [21]. In this regard, Horn and Horn [20] showed that child behaviour is determined by parents’ beliefs and value systems such as attitudes or values. In this regard, parents frequently react to their sons’ sports performance [22] even with aggressive behaviours toward coaches and referees [23]. These comments, which can range from supportive to more controlling [24], can increase parental pressure [25]. In this sense, parents’ attitudes vary according to the different stages of their children’s sports development. In the early years, parents tend to exhibit positive behaviours (supportive, emotionally intelligent discussions, and psychological and social development of the child), while in the middle stage, negative and critical behaviours emerge (excessive pressure, overemphasis on winning and talent development over other areas of the child’s life and use of controlling behaviours) [26]. This is relevant since when pressure increase, enjoyment and satisfaction decrease [27], leading to a demotivation for sports practice [25]. Pressure and demotivation would make those children to not be enrolled in sport or physical activity anymore. This is important due to the negative consequences, in terms of health, of sedentary behaviours [6].

Previous studies have analyzed the relationship between coaches, parents and athletes [28,29,30]. Holden, Forester, Keshock and Pugh [28] gave practical recommendations, such as educate parents on their responsibilities and expectations, to strengthen the relationships within the athletic triangle. Furthermore, Horne et al. [31] found poor communication, mistrust and lack of shared goals between coaches and parents. This attitude would compromise a child’s development. Lack of communication with coaches could be due to parents’ previous experience with sports activities or the team’s social environment [32]. In this regard, a previous study showed that if coaches’ relationships with parents were positive, the interest shown towards their children would be positively influenced. However, coaches perceive more negative behaviours than positive ones from parents [33,34,35]. Due to the relevance of parents in the children’s sports practice, a previous study designed and validated a questionnaire that measures coaches’ perceptions about the attitudes and behaviour of parents in sports. This questionnaire comprises three factors: the competition attitude, the communication with the coach, and the environment during training sessions [36].

Considering the relevance of the relationship between coaches and parents, it would be interesting to know the coach’s opinion regarding parents’ attitudes. Nevertheless, any previous study has focused on investigating coaches’ perception regarding parents’ attitude (in terms of competition, environment and communication), dividing coaches taking into account the type of institution, sex or sport modality. This analysis would determine which aspects are needed to be improved to reinforce the relationship between coaches and parents. Therefore, this study aimed to investigate the coach’s perception of parents’ attitudes regarding competition attitude, the communication with the coach and the environment during training sessions; taking into account the coaches´ sex, type of institution, or sport modality grouped into individual and collective sports. We hypothesized that: (a) the coaches of collective sports will perceive worse the relationship with the parents than the coaches of individual sports; (b) the coaches of clubs will perceive worse the relationship with parents, specifically, the competition factor, than coaches of public institutions; and (c) female coaches will have a worse relationship with parents than male coaches.

## 2. Materials and Methods

### 2.1. Study Design and Sample Size Estimation

A selective analytical and cross-sectional study was conducted [37]; since a questionnaire was used on a sample of participants, representative of a population, at a given time. Comparison between groups and explanation of behaviours was intended.

In order to estimate the sample size, the Free Statistic Calculator online resource (version 4.0) [38] was used. This online resource is based on the recommendation and formulas extracted from Cohen [39] and Westland [40]. Taking into account our CFA model, a total sample size of 156 participants was recommended.

### 2.2. Participants and Procedure

A total of 159 coaches of formative sports, at school-age, participated in this study (22 females, 13.84%; and 137 males, 86.16%). Inclusion criteria were: (a) school-age sports coaches; (b) be a coach of an individual or a collective sport; (c) to be at least five years of experience. Coaches were excluded if: (a) they did not meet inclusion criteria; (b) did not answer any of the questionnaire questions. In order to recruit participants, two of the researchers shared in social media (WhatsApp and Facebook) the evaluation instrument: Questionnaire for Sports Coaches about their Opinion on the Attitudes of Fathers and Mothers in Sports (CTPMD, Spanish acronym) [36]. This questionnaire was validated to obtain adequate content validity and internal consistency (Aiken’s *V* ≥ 0.81; *α* ≥ 0.63). It is composed of 19 items that measure three factors. Responses are given on a Likert-type scale from 1 to 5 points, where 1 = No, nothing/No, never and 5 = Yes, a lot/Yes, always [36]. The factors, considered as the dependent variables of the study, are:

(1) Competition attitude (items 1 to 7): to know the parent’s thoughts of the competition, i.e., importance they attach to winning, losing, and being a champion. For instance, item 1: For parents, their child’s winning is the most important thing.

(2) Communication (items 8 to 14): to know whether parents talk to coaches at training sessions and/or competition when the children win or lose. For instance, item 9: Parents talk to you at training sessions.

(3) Environment (items 15 to 19): to know the relationship of the parents with coaches in the training sessions, i.e., whether the positive (acceptance) or negative (anger) attitude. For instance, item 16: The parents’ environment with you, in general, is positive.

Interested people who met the inclusion criteria were contacted by a direct message or mail the researchers and completed the form. Then, they gave their consent to participate in the study and the publication of the results obtained. Once all the responses were received, the order of the scores in the environment factor was reversed. Therefore, five means the most negative value, and one means the most positive value in the three factors. Then, the fit of the factor structure proposed by the authors of the original scale and the reliability of the instrument was assessed (see outcomes section). Finally, statistical analysis of the data was performed.

The demographic variables of coaches’ sex (male/female), the institution type where they train (public/club), and the sport modality trained (collective/individual) were established as independent variables to find differences between groups.

Likewise, the study protocol respected the ethical guidelines of the Helsinki Declaration of 1975 and the Organic Law 3/2018, of 5 December, on the protection of personal data and guarantee of digital rights (BOE, 294, 6 December 2018), to guarantee the ethical considerations of scientific research with human subjects. The research was conducted through the approval of the Bioethics Committee of the University [approval number: September 2018].

### 2.3. Outcomes

We conducted a Confirmatory Factor Analysis (CFA) of the CTPMD instrument [36] to assess the model’s goodness of fit on each factor. The following fit indices were used: (a) the Chi-Square test of model fit. Non-significant chi-square values (*p* > 0.05) suggest a good fit of the model; (b) the Root Mean Square Error of Approximation (RMSEA). The values 0.01, 0.05 and 0.08 indicate excellent, good, and mediocre fit, respectively; (c) the Comparative Fit Index (CFI); (d) the Tucker Lewis Index (TLI) or Non-Normed Fit Index (NNFI); and (e) Normalized Fit Index (NFI). For the CFI, TLI/NNFI and NFI, the values greater than 0.90 indicate an acceptable fit, and those greater than 0.95 indicate an excellent fit [41]. The AMOS plugin (for SPSS 24.0 statistical software) was used for the CFA [42].

In such a way, it was necessary to eliminate item 4 for the factor’ competition attitude, items 10, 13, and 14 for the communication factor, and item 17 for the environment factor to obtain an excellent model fit (Table 1). Removing these items, Cronbach’s Alpha coefficient [43] showed acceptable values based on the number of items in each factor, according to Celia and Campo [44]: CTPMD *α* = 0.77; competition attitude *α* = 0.87; communication *α* = 0.71; environment *α* = 0.63. The number of items on a scale directly affects the value of Alpha. As the number of items increases, the variance of the numerator increases systematically [45]. The literature suggests 0.60 as the minimum acceptable value for Cronbach’s Alpha coefficient [46].

The Average Variance Extracted (AVE) and Composite Reliability (CR) were also calculated to analyze the reliability. The AVE is greater than 0.50 for the competition attitude factor and lower than 0.50 for the communication and environment factors. However, the CR is higher than 0.60 for the three factors (Table 1). Fornell and Larcker [47] stated that the convergent construct validity is still adequate if the CR is higher than 0.60, even if the AVE is lower than 0.50. Likewise, the AVE is close to the acceptable value of 0.40 in the communication and environment factors.

The demanding statistical process to obtain an excellent model fit and its reliability makes the study’s results very robust. These items (4, 10, 13, 14, 17) were removed before conducting comparisons between groups.

### 2.4. Statistical Analysis

Firstly, the criterion assumption tests (K-S, Rochas and Levene tests) were conducted to identify the hypothesis-testing model, which indicated the use of non-parametric mathematical tests.

Then, a descriptive analysis was performed using the mean and standard deviation. The Mann-Whitney U test was also calculated to analyze the differences between the categories of the independent variables studied for each of the factors [48].

Finally, the effect size was calculated using the Rosenthal r formula [49]. Therefore, the criteria proposed by Cohen [39] were assumed: 0.10 to 0.30, small effect; 0.30 to 0.50, intermediate effect; and 0.50 and higher, strong effect.

SPSS 21.0 statistical software (IBM Corp. Released 2012. IBM SPSS Statistics for Windows, Version 21., IBM Corp, Armonk, NY, USA) was used for the statistical analyses. The significance level was set at *p* < 0.05.

## 3. Results

None of the recruited participants was excluded from statistical analyses. Table 1 shows the CFA, AVE, and CR results for the three factors, competition attitude, communication and environment. Results indicate an excellent model fit for each factor after eliminating the items indicated by the CFA: *X*^2^ > 0.05; RMSEA = value < 0.05; CFI = value > 0.95; TLI/NNFI = value > 0.95; NFI = value > 0.95. The reliability is also adequate with CR values higher than 0.60 for each factor.

**Figure 1:**
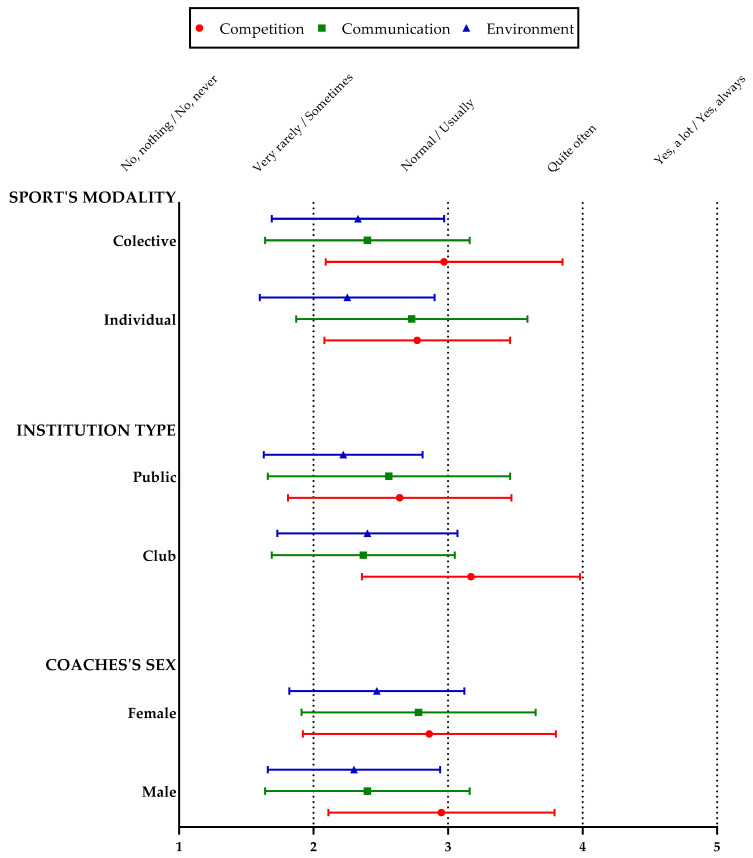
Descriptive results of the independent variables studied for the factors competition attitude, communication with the coach and environment during training sessions.

Descriptive results are depicted in Figure 1. The value 5 indicates the most negative value, and one means the most positive value in the three factors. The means indicate that those male coaches who train collective sports and in clubs perceived a greater parent’s competition attitude. Coaches-parent communication is more negative perceived by female coaches who train individual sports and in public institutions. Likewise, female coaches who train collective sports and in clubs perceived a more negative environment from parents. Based on these descriptive results, it was necessary to perform an inferential analysis to identify statistically significant differences between the categories of the independent variables studied (Table 2).

Table 2 shows the differences between the categories of the independent variables studied. There are significant differences in the competition attitude factor according to the type of institution in favour of public institutions (*U* = 1964.500; *p*-value < 0.001; *r* = 0.31). Likewise, there were significant differences in the communication factor according to the coaches’ sex in favour of male coaches (*U* = 1112.000; *p*-value = 0.04; *r* = 0.16).

## 4. Discussion

Parental attitudes in sports can significantly influence the behaviour of coaches and the sport adherence of young athletes. This study’s originality lies in the coach’s perception of parents’ attitudes regarding competition attitude, communication with the coach, and environment during training and/or competition. In this regard, this is the first study that analyzes the coach’s perception taking into account their sex, type of institution and sport modality (individual and collective sports) that they train. We hypothesized that: (a) the coaches of collective sports will perceive worse the relationship with the parents than the coaches of individual sports; (b) the coaches of clubs will perceive worse the relationship with parents, specifically, the competition factor than coaches of public institutions; and (c) female coaches will have a worse relationship with parents than male coaches. Therefore, the key findings of this article are: (1) differences were not found when compared individual to collective sports coaches´ perception, (2) coaches of clubs perceived the competition attitude higher than coaches who work for public institutions, and (3) females coaches showed lower values of communication with parents than males’ coaches.

For the first time, our results showed that female coaches perceived less positive communication with parents than male coaches. Overall, Yabe et al. [32] identified that among the respondents, 29% of parents felt a lack of communication with their children’s coaches. Previous studies showed that gender could impact the perception of leadership in sport contexts [50]. In this sense, Manley et al. [51] reported that athletes perceive female coaches as less competent than male coaches. Furthermore, Magnusen and Rhea [52] showed that male athletes were more comfortable with male coaches, exhibiting negative attitudes towards female coaches. This aligns with coaches’ opinions since female coaches perceived that male athletes continuously test them [53]. Interestingly, Lorimer and Jowett [54] have demonstrated that a male coach working with female athletes reinforced the traditional gender and sport role. Hypothetically, this genre stereotype could be extrapolated to the relationship between parents and coaches. This could explain why female coaches perceived less positive the communication with parents. Nevertheless, Murray, Lord and Lorimer [50] indicated that female coaches were rated higher in terms of relationship quality and empathy than male coaches. Therefore, future studies should explore this interesting topic.

In Europe, children have two main options to practice sport in their leisure time: sports clubs or public institutions [12]. In this regard, our results showed that club coaches perceived a higher competition attitude than coaches who work for public institutions. This finding could be supported by the non-significant trend observed in the environment factor, where higher values were obtained in the club coaches’ perception. In this regard, a more negative environment may result from parents’ overemphasis on doing well in clubs. This may be due to that clubs pursue a higher performance-oriented politics compared to public institutions. This could be relevant since stress, anxiety, or pressure negatively impacted the adherence to physical activity in children and adolescents [55,56]. Although significant differences were not found when analyzing the sport modality (individual or collective sport), collective sports coaches perceived a higher competition attitude than coaches of individual sports. This could be explained since parents could pressure coaches to choose their son/daughter to increase their participation during competitions. In this regard, coaches expressed discomfort with parents for their intromission in the coaches’ work [35]. Nevertheless, this hypothesis should be explored in future studies due to the importance of these aspects.

Practising sports is one of the main options that children consider in their leisure time [10,11]. In this regard, a previous study that analyzes leisure activities among German children and adolescents estimates that 53% of adolescents´ boys and 33% of adolescents´ girls spend time doing sports. However, preschool and primary school-age children showed higher participation rates, ranged from 56% among boys and 54% among girls. Adherence to these active habits is quite relevant since physical activity enrolment during childhood is of great relevance due to the benefits on social [2], mental [3], and physical health [4]. Furthermore, regular practice could decrease the risk of chronic diseases [5] such as obesity [6,7] or even ameliorate the negative impact of the COVID-19 pandemic on the quality of life of children and adolescents [9]. Nevertheless, the parents’ behaviour plays an important role in the children’s sports development and value formation [19,23]. Furthermore, the motivational climate of parents can influence how children understand, perceive and react to achievement such as competitions or training [20]. Regarding parents attitudes, previous studies have documented that it is common to find parents reacting to their sons’ sports performance [22] even with aggressive behaviours to coaches and referees [23]. In this regard, Shields, Bredemeier, LaVoi and Power [23] showed that 13% of analyzed parents acknowledged having angrily criticized their child’s sports performance. In the same line, 15% of children reported that their parents get angry when they do not do well. Moreover, 21% of children and adolescents prefer that their parents stay home rather than watch the competition. These results could explain the high level of children and adolescents who drop-out of sport [57].

This study has some limitations that should be acknowledged. First, a higher number of male coaches than female coaches participated in this cross-sectional study. A higher percentage of male coaches could explain this than female coaches working in the out-of-school context [58]. Nevertheless, future studies should include further female coaches in order to explore if the differences obtained in our study are still observed. Second and in the same line as the first limitation, the number of individual sport coaches is lower than collective sports coaches. However, this study also has strengths. In this regard, to the best of our knowledge, this is the first study, which analyzes and compares the impact of sex on the perception of parents’ attitudes. Therefore, results would highlight the genre stereotype, explaining the difference in parents’ communication attitudes.

This study allows learning about parents’ attitudes regarding the sports practice of their children. This is relevant to determine whether parents want an educational and recreational sport that helps to improve their children’s integral development or, on the contrary, a more oriented-competition sport, which could generate abandonment of sports practice. As a practical recommendation, starting the season with a parents meeting would be crucial to improve the school-age sport. This meeting would allow bringing the expectations of parents, athletes, and coaches closer together. As Holden, Forester, Keshock and Pugh [28] recommended, this information must be communicated and understood. Thus, parents, players, and coaches should sign the contract to understand and accept the information provided. The signing of the behaviour contracts might be useful as a reminder later in the season if any conflict emerges. Educating parents about youth sports so that appropriate communication between coaches and parents will enhance athlete development [19]. Furthermore, and taking into account our results, parents should be trained in the three factors studied (competition attitude, communication, and environment) in the initial meeting to make them understand the need to create a positive climate with the coach [33] and the importance of the educational character that sport must acquire in the training stages, including sport clubs. Moreover, coaches should also be trained to react to parents’ attitudes. This training will generate a positive coach-parent relationship that will benefit the children as it will encourage greater sports adherence with improvements in health-related quality of life. This training would improve the deficit information which has been detected in Spain’s coaches [59].

## 5. Conclusions

For the first time, this study showed that females’ coaches perceived lower values of communication with parents than males’ coaches, which could be due to gender stereotypes. The originality of this study also lies in the fact that we found that club coaches perceived a higher competitive attitude than coaches working for public institutions. This could be relevant since pressure negatively impacted the adherence to physical activity in children and adolescents.

## Figures and Tables

**Table 1:** CFA, AVE, and CR of the evaluation instrument CTPMD.

Factor	*X* ^2^	RMSEA	CFI	TLI/NNFI	NFI	Fix Model	AVE	CR
Competition	0.32	0.03	1.00	0.99	0.98	Excellent	0.59	0.90
Communication	0.92	0.00	1.00	1.00	1.00	Excellent	0.38	0.70
Environment	0.35	0.00	1.00	1.00	1.00	Excellent	0.32	0.63

Note: *X*^2^ = *p*-value of the Chi-Square test; RMSEA = Root Mean Square Error of Approximation; CFI = Comparative Fit Index; TLI = Tucker Lewis Index; NNFI = Non-Normed Fit Index; NFI = Normed Fit Index; AVE = Average Variance Extracted; CR = Composite Reliability.

**Table 2:** Differences between the categories of the independent studied variables.

Variable	Categories	Competition *M* (*SD*)	Communication *M* (*SD*)	Environment *M* (*SD*)
Sport´s modality	Individual (*n* = 23)	2.77 (0.69)	2.73 (0.86)	2.25 (0.65)
	Colective (*n* = 135)	2.97 (0.88)	2.40 (0.76)	2.33 (0.64)
	*U*	1305.500	1215.00	1482.00
	*p*-value	0.22	0.09	0.73
	*R*	0.10	0.13	0.03
Type of institution	Club (*n* = 91)	3.17 (0.81)	2.37 (0.68)	2.40 (0.67)
	Public (*n* = 68)	2.64 (0.83)	2.56 (0.90)	2.22 (0.59)
	*U*	1964.500	2802.500	2552.500
	*p*-value	<0.001 *	0.31	0.06
	*r*	0.31	0.08	0.15
Coaches’s sex	Male (*n* = 137)	2.95 (0.84)	2.40 (0.76)	2.30 (0.64)
	Female (*n* = 22)	2.86 (0.94)	2.78 (0.87)	2.47 (0.65)
	*U*	1422.500	1112.000	1247.000
	*p*-value	0.67	0.04 *	0.19
	*r*	0.03	0.16	0.10

Note: *n* = Sample; *M* = Mean; *SD* = Standard Deviation; *U* = Mann-Whitney U test; *r* = Rosenthal r; * *p* < 0.05.

## Data Availability

Data will be available upon reasonable request to the corresponding author.
